# Improving applicability of the new obesity classification based on weight history in severe obesity

**DOI:** 10.20945/2359-3997000000572

**Published:** 2022-11-28

**Authors:** Flávia Galvão Cândido, Olívia Gonçalves Leão Coelho, Karla Pereira Balbino, Helen Hermana Miranda Hermsdorff

**Affiliations:** 1 Universidade Federal de Viçosa Departamento de Nutrição e Saúde Viçosa MG Brasil Departamento de Nutrição e Saúde, Universidade Federal de Viçosa, Viçosa, MG, Brasil; 2 Universidade Federal de Viçosa Instituto de Políticas Públicas e Desenvolvimento Sustentável Viçosa MG Brasil Instituto de Políticas Públicas e Desenvolvimento Sustentável, Universidade Federal de Viçosa, Viçosa, MG, Brasil

**DEAR EDITOR,**

We read with great enthusiasm the recently published Brazilian proposal for obesity classification based on weight history (1). In fact, it established a target for success in non-surgical obesity treatment. However, we would like to raise some discussions regarding its applicability in severe obesity.

Adults living with severe obesity present specific challenges during clinical management of weight, what makes those unresponsive considered for bariatric surgery. At the same time as they are more likely to present weight reductions (2), they usually need greater weight loss to be healthier (3–5) and have difficulties in sustaining weight loss, culminating in greater instability in their weight trajectories (3). Furthermore, attaining normal weight during clinical treatment is rarer in severe obesity than in simple obesity (2), which increases the need to set specific weight loss goals for these individuals.

International guidelines which have referred to specific goals for severe obesity (3,4) present bolder proposals in relation to BMI range and the respective goals, compared to the Brazilian proposal (1). While Brazilian proposal adopts generalist goals (5%-10%) for BMI between 30.0 to 40.0 kg/m^2^ and recommends greater weight loss (10%-15%) only for BMI ≥ 40.0 kg/m^2^ (1), international guidelines advice greater weight loss (15%-20% or even > 20%) already for BMI from 35.0 kg/m^2^ (3,4). According to the Brazilian classification (1), individuals with a BMI between 35.0 to 39.9 kg/m^2^ present obesity-controlled, while they are good responders to non-surgical treatment if they reach a weight loss greater than 10%. However, this could not represent real improvement in general health condition, especially for metabolic unhealthy individuals.

Another discussion point is that the Brazilian proposal incorporated only BMI into their classification and international guidelines presuppose increased goals based not only on BMI, but also on the severity of the condition identified by the presence of comorbidities (4,5). This seems to make Brazilian classification simpler, but less accurate. Otherwise, disregarding the presence of comorbidities may impair its applicability during evaluation for bariatric surgery, once worldwide eligibility criteria include failure in non-surgical treatment for BMI between 35.0 to 40.0 kg/m^2^ with comorbidities or BMI between 40.0 to 50.0 kg/m^2^. Thus, proposed a double success classification criterion for these individuals does not seem to be the best alternative.

In conclusion, we suggest a simple adaptation of the Brazilian proposal (1) represented by an asterisk (“*”) aside the BMI range of 30.0 to 40.0 kg/m^2^ in “Table 1”, followed by a footnote saying: “for individuals with BMI between 35.0 to 39.9 kg/m^2^ and comorbidities, consider the use of the same ranges of those with BMI from 40.0 to 50.0 kg/m^2^”. Besides, the ranges values in that table could be better defined (e.g. replace “40.0” by “39.9” in the first line, “>10” by “≥10” and “>15” by “≥15”). This adapted classification can be a useful and suitable alternative to guide clinical management of adults living with severity obesity.
